# Implication and Regulation of AMPK during Physiological and Pathological Myeloid Differentiation

**DOI:** 10.3390/ijms19102991

**Published:** 2018-09-30

**Authors:** Arnaud Jacquel, Frederic Luciano, Guillaume Robert, Patrick Auberger

**Affiliations:** 1 Université Côte d’Azur, C3M Inserm U1065, 06204 Nice, France; jacquel@unice.fr (A.J.); fluciano@unice.fr (F.L.); robertg@unice.fr (G.R.); 2 Equipe Labellisée par la Fondation ARC, 94803 Villejuif, France

**Keywords:** AMPK, monocytes, macrophages, differentiation, autophagy, AML, MDS, CML, CMML

## Abstract

AMP-activated protein kinase (AMPK) is a heterotrimeric serine/threonine kinase consisting of the arrangement of various α β, and γ isoforms that are expressed differently depending on the tissue or the cell lineage. AMPK is one of the major sensors of energy status in mammalian cells and as such plays essential roles in the regulation of cellular homeostasis, metabolism, cell growth, differentiation, apoptosis, and autophagy. AMPK is activated by two upstream kinases, the tumor suppressor liver kinase B1 (LKB1) and the calcium/calmodulin-dependent protein kinase kinase 2 (CAMKK2) through phosphorylation of the kinase on Thr172, leading to its activation. In addition, AMPK inhibits the mTOR pathway through phosphorylation and activation of tuberous sclerosis protein 2 (TSC2) and causes direct activation of unc-51-like autophagy activating kinase 1 (ULK1) via phosphorylation of Ser555, thus promoting initiation of autophagy. Although it is well established that AMPK can control the differentiation of different cell lineages, including hematopoietic stem cells (HSCs), progenitors, and mature hematopoietic cells, the role of AMPK regarding myeloid cell differentiation is less documented. The differentiation of monocytes into macrophages triggered by colony stimulating factor 1 (CSF-1), a process during which both caspase activation (independently of apoptosis induction) and AMPK-dependent stimulation of autophagy are necessary, is one noticeable example of the involvement of AMPK in the physiological differentiation of myeloid cells. The present review focuses on the role of AMPK in the regulation of the physiological and pathological differentiation of myeloid cells. The mechanisms of autophagy induction by AMPK will also be addressed, as autophagy has been shown to be important for differentiation of hematopoietic cells. In addition, myeloid malignancies (myeloid leukemia or dysplasia) are characterized by profound defects in the establishment of proper differentiation programs. Reinduction of a normal differentiation process in myeloid malignancies has thus emerged as a valuable and promising therapeutic strategy. As AMPK seems to exert a key role in the differentiation of myeloid cells, notably through induction of autophagy, we will also discuss the potential to target this pathway as a pro-differentiating and anti-leukemic strategy in myeloid malignancies.

## 1. Introduction

AMPK is an essential regulator and sensor of cellular energy status in mammalian cells. This kinase integrates changes in the AMP/ATP and ADP/ATP ratios, fine-tuning the balance between ATP consumption and synthesis [1,2]. AMPK acts to increase the rate of catabolic process and to decrease the rate of anabolic process to sustain intracellular energy homeostasis [3]. AMPK is activated by a wide range of stimuli, such as nutrient deprivation [4], cellular stresses [5], fasting or caloric restriction [6], caloric restriction mimetics [7,8,9,10], and nucleoside analogues such as (5-aminoimidazole-4-carboxamide-1-β-d-ribofuranoside; AICAR) [11], and has been evaluated in clinical trials for the treatment of metabolic diseases and cancers, including both hematopoietic malignancies and solid tumors [12,13,14,15]. In addition to its well-established role in maintaining energy homeostasis, AMPK is also extensively involved in promoting autophagy [16,17,18], longevity, and tumor suppression [19,20,21,22].

AMPK is a highly conserved heterotrimeric serine/threonine kinase that phosphorylates a plethora of cellular substrates implicated in numerous metabolic pathways through rapid or long-term adaptive responses to different metabolites [23]. The first thoroughly characterized AMPK substrate was acetyl-CoA-carboxylase, which catalyzes the carboxylation of acetyl-CoA to produce malonyl-CoA, highlighting an essential role for this kinase in the induction of fatty acid oxydation [24]. More generally, AMPK intervenes at the crossroad of different key cellular signaling pathways, monitoring cellular energy status, cell proliferation, differentiation and autophagy, notably through its negative control of the PI3K/AKT/mTOR pathway and its stimulatory effect on autophagy through direct phosphorylation of ULK1 (ATG1, a serine threonine kinase involved in the initiation of this catabolic process) [25,26,27]. AMPK negatively regulates energy-consuming cell proliferation/growth, whereas it induces an energy compensating program, such as autophagy. It is well established that AKT signaling favors glucose uptake and glycolysis, therefore increasing ATP production and reducing AMPK activation. To that end, AKT directly phosphorylates AMPK; on ser487, hindering the activation of AMPK by LKB1 [28]. AKT signaling through mTORC1 stimulates ATP-consuming anabolic processes, whereas AMPK blocks anabolic metabolism to favor catabolic processes. In addition, AKT and AMPK exert opposing effects on mTORC1 signaling as well as nutrient and glycogen synthesis. In addition to regulating cell proliferation, AKT, through mTORC1, inhibits autophagy. Indeed, ULK1 activity is repressed by mTORC1 complex leading to autophagy inhibition. By contrast, phosphorylation of TSC2 by AMPK increases its activity, leading to mTORC1 inhibition and indirectly to autophagy induction. Finally, AMP directly phosphorylates ULK1 on ser555 to promote induction of autophagy. In conclusion, cross-talk between these two kinases is critical to switch from the catabolic to the anabolic states (and vice versa) and to balance autophagy [29].

Recent findings also indicate that AMPK can sense glucose availability independently of adenine nucleotide binding through the formation of a complex with axin at the lysosomal membrane [30,31]. Finally, in addition to its essential role in the regulation of cellular metabolism and autophagy, AMPK also plays a critical role in the regulation of anti-inflammatory responses [32,33] and cell growth and differentiation [4,16,34]. In this context, modulation of the AMPK pathway has emerged as a promising strategy in a wide range of human pathologies, including metabolic and inflammatory diseases and cancer.

There is increasing evidence that AMPK is also required for many types of cellular differentiation processes by providing through different mechanisms (increased metabolism and activation of autophagy, for instance) the fuel and nutrients (glucose, amino acids, lipids, and sugars) that sustain the modification of the transcription programs necessary for the completion of cellular differentiation. The present review emphasizes the role of AMPK in the differentiation of hematopoietic cells, with a special focus on the physiological and pathological differentiation of myeloid cells. The opportunity to use recently developed AMPK activators for the treatment of myeloid malignancies, including myelodysplastic syndrome (MDS), acute myeloid leukemia (AML), chronic myelogenous leukemia (CML), and chronic myelomonocytic leukemia (CMML) will also be addressed.

## 2. AMPK Structure and Activation

**Figure 1:**
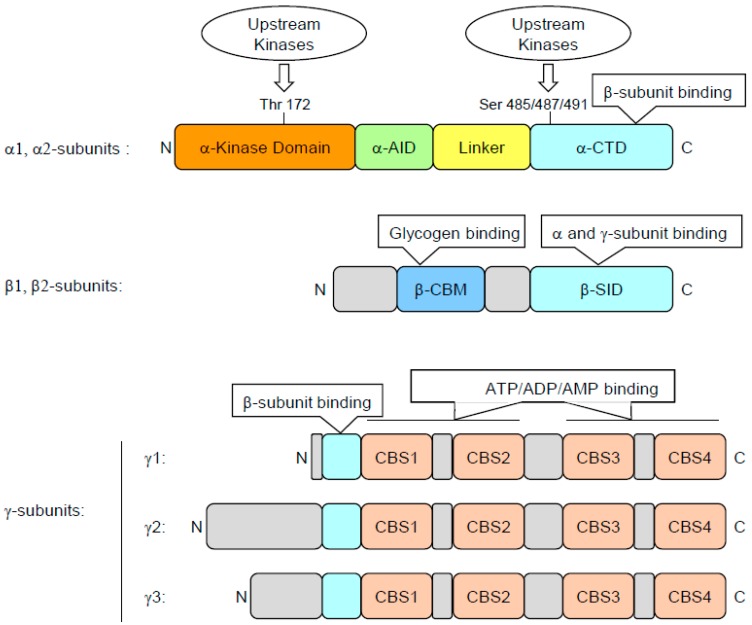
Structure of mammalian AMPK subunits. AMPK is a heterotrimeric protein consisting of 1 catalytic subunit (α subunit) and 2 regulatory subunits (β and γ subunits). The α subunit contains a kinase domain (α-KD), the activity of which relies on the phosphorylation of Thr172 by upstream AMPK kinases. The kinase domain is followed by an autoinhibitory domain (α-AID) that is joined to the COOH-terminal domains (α-CTD) by a less well conserved linker. The α-CTD domain binds to the C-terminal domain of the β subunit. The β-subunit contains two conserved regions: (1) a carbohydrate-binding module (β-CBM) that causes the mammalian complex to bind to glycogen particles and (2) a COOH- terminal subunit interaction domain (β-SID) that provides the bridge between the α- and γ-subunits. The γ-subunit contains variable NH2-terminal regions followed by a short sequence involved in binding to the β-subunit and by four tandem repeats of a cystathionine-β-synthase (CBS) motif that act in pairs to form the binding sites for adenine nucleotides (ATP, ADP and AMP).

The structure and activation modalities of AMPK have been excellently reviewed recently [34,35]. Briefly, the AMPK catalytic subunits (α1 and α2) are encoded by two different genes called PRKAA1 and PRKAA2. The α subunits consist, from their N- to C-termini, of N- and C-lobes, which together form the kinase domain (α-KD) typical of serine/threonine kinases, an auto-inhibitory domain (α-AID), an α-linker domain, and finally a globular C-terminal domain (α-CTD). The scaffolding beta subunits (β1 or β2) are encoded by two different genes (PRKAB1 and PRKAB2). They display a myristoylated N-terminal region involved in the recruitment of AMPK to the membrane of mitochondria or phagophores, followed by a central carbohydrate-binding module domain (β-CBM) and a C-terminal subunit interaction domain (β-SID). The γ regulatory subunits (γ1, γ2, or γ3) are encoded by three different genes: PRKAG1, PRKAG2, and PRKAG3. The γ subunits are characterized by an N-terminal region of variable length and the presence of four tandem cystathionine-beta-synthase repeats (CBS1 to CBS4), that are involved in the binding of the regulatory nucleotides AMP, ADP, and ATP (Figure 1). The α, β, and γ AMPK subunits assemble into different heterotrimeric serine/threonine kinases depending on their tissue distribution and on the representativeness of the various isoforms (Figure 1). In myeloid cells, the α1β1γ1 complex is predominantly expressed. Of note, AMPK complexes composed of different gamma subunit isoforms (γ1 to γ3) exhibit exquisite variations in their sensitivity to increased AMP and ADP levels that enable subtle variations in the regulation of the kinase activity. This suggests that AMPK could respond differently and selectively to changes in adenine nucleotide depending on the nature of the γ subunit present in the complex [36]. AMPK is activated by phosphorylation at Thr172 in the C-lobe domain, an event that is achieved by one of the three main upstream kinases, the tumor suppressor LKB1, CaMKK2, or TAK1 [37]. Other regulatory sites, such as Ser487 in the α1 subunit and Ser491 in the α2 subunit, that are phosphorylated by either protein kinase A, protein kinase B (AKT), or ribosomal S6 kinase are implicated in the negative regulation of AMPK and thus favor activation of the mTOR pathway. Once activated, AMPK phosphorylates a plethora of protein substrates that are involved in the regulation of cell energetic metabolism, autophagy, modulation of proliferation, regulation of inflammatory responses, and differentiation [38]. Thus far, more than 50 AMPK substrates have been identified in mammalian cells that are involved in energy homeostasis but also in other functions not directly linked to metabolism regulation [39]. However, the phosphorylome of AMPK is far from completely known, as recently attested by the identification of TET2 (Tet-eleven translocation 2) phosphorylation by AMPK linking diabetes to cancer [40].

## 3. AMPK and Differentiation of Hematopoietic Stem Cells

Hematopoietic stem cells (HSCs) are multipotent, self-renewing progenitor cells that can replenish all blood cell types in a process called “hematopoiesis.” HSCs differentiate into two lineage-restricted, lymphoid and myelo-erythroid, oligopotent progenitor cells that will ultimately give rise to lymphocytes, granulocytes, macrophages, erythrocytes, and platelets. An alternative, “myeloid-based” model for blood lineage differentiation also implies a common myelo-lymphoid progenitor cell that generates progeny from both lineages. The mechanisms controlling HSC self-renewal and differentiation are influenced by a diverse set of cytokines, chemokines, receptors, and intracellular signaling molecules. Producing 500 billion cells a day from a limited number of HSCs is a highly energy-consuming process. How hematopoietic stem cells (HSCs) accommodate their energy requirement during growth and differentiation stages remains poorly understood. Recent studies in the literature have established that the LKB1 tumor suppressor, one of the AMPK upstream kinases, is critical for the maintenance of energy homeostasis in HSCs. In different studies, *Lkb1* invalidation in mice has been shown to cause loss of HSC quiescence, division, rapid depletion, and pancytopenia [41]. In addition, *Lkb1*-deficient bone marrow cells display drastic mitochondrial defects, defaults in lipid and nucleotide metabolism, and, as a consequence, decreased ATP consumption. Nevertheless, AMPK seems to exert only a marginal role in HSC depletion. While HSC exhaustion appears to occur largely independently of AMPK, the defects in mitochondrial function are also observed in *Ampk*-deficient mice [41], suggesting that AMPK can act in concert with LKB1 to regulate mitochondrial fitness but not HSC depletion. These data highlighted an essential role for LKB1 as an inhibitor of HSC proliferation but a likely more minor role of AMPK and mTOR in this process [42]. As mentioned previously, the LKB1/AMPK pathway is one of the main activators of autophagy [25,43]. It is therefore possible that the changes in mitochondrial mass and decreased ATP levels observed in the absence of LKB1 and AMPK could reflect alteration in the rates of autophagy in deficient mice. 

Differentiation of HSCs into their different progeny, including myeloid precursors, requires high energy status levels. Recent studies have highlighted the crucial role of mitochondrial oxidative phosphorylation for the differentiation of HSCs. Indeed, specific invalidation of the PTEN-like mitochondrial phosphatase PTPMT-1 in HSCs resulted in an increase of the HSC pool and a block in differentiation [44]. Importantly, reintroduction of catalytically active PTPMT1, but not catalytically inactive PTPMT1 or PTPMP1 lacking mitochondrial localization signal, restores the differentiation capabilities of PTPMT1-deficient HSCs. This blockade in differentiation potential of HSCs is linked to altered mitochondrial metabolism in the absence of PTPMT1. While PTPMT1 seems to be essential for HSC commitment, depletion of PTPMT1 in myeloid or lymphoid progenitors failed to cause any defect in lineage-specific knock-out mice. It is likely that the altered mitochondrial metabolism caused by *PTPMT1* deficiency is sensed by AMPK and then relayed to the p53-p21/p57 pathway, as p21 and p57 expression was significantly upregulated in PTPMT1 depleted stem cells and early progenitors. AMPK on its own could exert its effect on mitochondria through phosphorylation and activation of PGC-1α and β, which are master transcriptional regulators of mitochondrial biogenesis [45].

## 4. AMPK and Physiological Monocyte Differentiation

**Figure 2:**
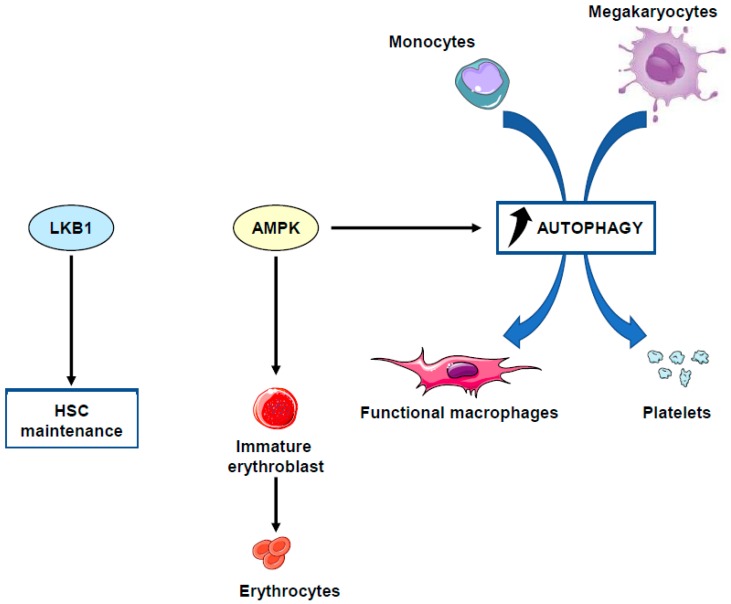
Role of LKB1 and AMPK in HSC maintenance and hematopoietic cell differentiation. There is genetic evidence that LKB1 is required for HSC maintenance since *Lkb1* depletion in mice results in loss of HSC quiescence, division and rapid depletion that contributes to pancytopenia. AMPK for its own is required for the early steps of erythroid differentiation, the production of functional macrophages from monocytes and the differentiation of HSCs into megakaryocytes and ultimately functional platelets.

**Figure 3:**
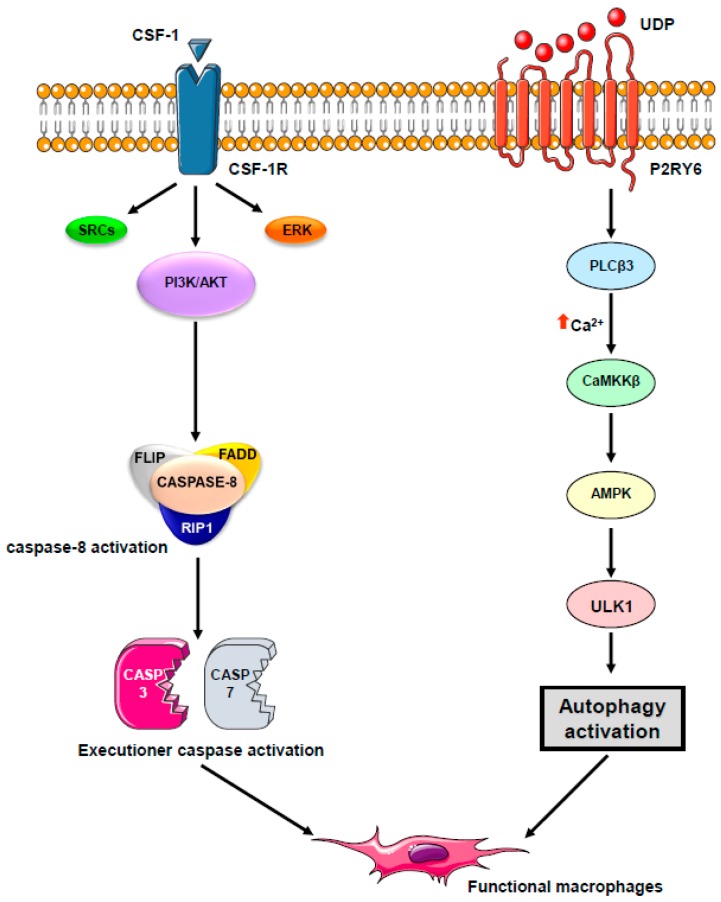
A schematic molecular view of the pathway involved in macrophagic differentiation. Engagement of the CSF-1 tyrosine kinase receptor by CSF-1 activates the PI3K/AKT pathway and induces caspase-8 activation within a FADD/RIP/FLIP multimolecular complex. In turn, active caspase-8 triggers a spatially restricted activation of caspase-3 and -7 that cleave selected intracellular proteins to generate a resting macrophage phenotype. Engaged CSF-1R also promotes autophagy through increasing the expression of the purinergic receptor P2RY6 that activates the CAMKK2-AMPK-ULK1 pathway. Caspase activation and autophagy induction are both required for proper generation of macrophages upon CSF-1 stimulation.

Beyond their originally described role as conveyors of programmed cell death and inflammation, caspases are involved in a number of other cellular functions, the most prominent being cell differentiation, a conserved property across a plethora of cell types in divergent metazoan organisms (Figure 2). Caspases mediate DNA damage and morphological changes that are common to different cell fates. The specificity of their action may be controlled by a diversity of mechanisms, including protein–protein interactions, post-translational modifications, subcellular localization, and interaction with other fundamental cell processes such as autophagy and proteostasis. Macrophages, which are mainly derived from monocytes, are essential components of mammal tissue homeostasis. Some are seeded into tissues before birth, while others are continually replenished from blood monocytes. Monocytes are circulating blood leukocytes [46] that migrate into tissues where they differentiate into morphological and functionally heterogeneous cells, including macrophages, myeloid dendritic cells, and osteoclasts [47]. Their differentiation can be recapitulated ex vivo by incubation with cytokines, e.g., they differentiate into macrophages upon exposure to colony stimulating factor-1 (CSF-1) [48]. The biologic effects of CSF-1 are typically mediated by plasma membrane associated CSF-1R [49]. Downstream signaling pathways include PI3K-AKT and AMPK, which mediate caspase activation and autophagy, respectively [50,51]. We have depicted these two pathways in more detail. Monocyte differentiation triggered by CSF-1 receptor (CSF-1R) engagement is critically dependent on the oscillatory activation of the kinase AKT, leading within 2–3 days to the formation of a multi-molecular platform. This molecular complex includes the adaptor molecule Fas-associated death domain (FADD), the serine-threonine kinase receptor-interaction protein kinase1 (RIP1), the long and short isoforms of flice inhibitory protein (FLIP), and procaspase-8 [52,53]. In turn, active caspase-8 provokes a spatially restricted activation of caspase-3 and -7 that cleaves selected intracellular proteins to generate a resting macrophage phenotype. This proteolytic machinery is inhibited as soon as macrophages are activated with lipopolysaccharides, a condition that also impairs autophagy induction. Engaged CSF-1R also promotes autophagy [50] through increasing the expression of the purinergic receptor P2RY6 that activates the CAMKK2-AMPK-ULK1 pathway [51]. Caspase activation and autophagy induction are both required for proper generation of macrophages upon CSF-1 stimulation (Figure 3 provides a schematic view of signaling network involved). The molecular links between caspase activation and autophagy require further investigation. One possible hypothesis is that post-translational modifications of caspases and differentiation-specific cleavage sites in cellular protein targets may be instrumental in connecting caspases to autophagy in cells undergoing differentiation. Thus, the CAMKK2/AMPK/ULK1 axis appears essential for proper differentiation of monocytes into macrophages. 

Of note, in freshly isolated monocytes there is limited if any expression of AMPK subunit proteins. During CSF-1-mediated differentiation of monocytes into macrophages, there is a time-dependent increase in AMPKα1 expression and phosphorylation on Thr172 and a concomitant increase in ULK1 level and phosphorylation on Ser555 [51]. Increase in AMPKα1 and ULK1 expression at the protein but not the mRNA level, by a still unknown mechanism, does not rely on inhibition of the proteasome activity. This increase in AMPKα1 protein expression is further accompanied by elevated phosphorylation and activation of AMPK and ULK1 and correlates with the induction of autophagy and myeloid differentiation.

Other evidence for AMPK implication in myeloid cell differentiation comes from additional experiments performed in U937 and HEL acute myeloid leukemia (AML) cell lines, in which low doses of cytarabine were found to trigger the autophagy necessary for myeloid differentiation, as shown by increased expression of CD11b and morphologic features of differentiation. Inhibition of the mTOR pathway, likely independent of AMPK, was associated with cytarabine-mediated myeloid differentiation [54]. A last example of a role for AMPK in autophagy-mediated differentiation stems from in vivo experiments conducted with AMPKα1 mice. Indeed, AMPKα1 deficiency was shown to impair autophagy-mediated differentiation and decrease monocyte macrophage survival [55]. Finally, α1 and α2 AMPK isoforms appeared to display functional differences in the regulation of osteogenesis and osteoblast-associated induction of osteoclastogenesis [56].

## 5. The Role of AMPK in Hematological Cancers

AMPK has been reported to inhibit the growth of various hematological cancers. Indeed, in acute lymphoblastic leukemia (ALL) cell lines, AICAR triggered dose- and time-dependent inhibition of cell growth [57,58]. The pro-apoptotic effect of AMPK is mediated by activation of the p38 MAPK pathway, increased expression of cell-cycle inhibitors such as p27 and p53, and the downstream effects of the mTOR pathway. In B-cell chronic lymphocytic leukemia (B-CLL) cells, AMPK triggered apoptosis in a p53-independent manner [13]. In mantle cell lymphoma (MCL), AICAR-mediated stimulation of AMPK activity dampened phosphorylation of critical downstream effectors of mTOR signaling, such as 4E-BP1 and ribosomal protein S6, leading to cell growth inhibition [59]. In chronic myeloid leukemia (CML) and Philadelphia chromosome positive ALL, metformin and AICAR suppressed the mTOR activity and cell growth [58,60]. In myelodysplastic syndromes (MDS) and some acute myeloid leukemia (AML) cell lines, AICAR induced suppression of cell growth independently of apoptosis induction and AMPK activation [12]. Globally, indirect activators of AMPK induced suppression of cell growth and activation of cell death through both AMPK dependent and independent mechanisms in different hematological malignancies.

## 6. AMPK in the Regulation of Pathological Myeloid Differentiation

### 6.1. Myelodysplastic Syndromes

Myelodysplastic syndromes (MDS) constitute a set of myeloid progenitor cell diseases characterized by an ineffective production of mature and differentiated myeloid cells. This pathology strongly impacts the elderly, with an incidence of 20/100,000 people affected at 60 years and up to 50/100,000 at 80 years. This heterogeneous pathology is a multistep disease, the first stage being characterized by the appearance of abnormal cells in the bone marrow that are unable to differentiate and therefore to produce mature and functional cells in peripheral blood. At this stage, the disease is considered low-risk MDS and characterized by excessive apoptotic cell death of CD34-positive cells. In the next stage, CD34-positive progenitors accumulate mutations and hypermethylation of their DNA [61]. These genetic and epigenetic modifications render MDS cells less sensitive to apoptotic signals and contribute to abnormal cell growth. At this stage, the disease is defined as high-risk MDS and associated with an increase in medullar blast count, a higher probability for transformation to AML and a worse prognosis. Each stage of the disease is correlated with survival expectancy and probability for AML transformation by the Revised—International Prognostic Scoring System (IPSS-R). For classification of patients, IPSS-R takes into account the percentage of medullar blasts, the number of cytopenias and the cytogenetic status [62]. The recommended treatment in lower-risk MDS is the management of transfusion needs, whereas in high-risk MDS and AML, the only curative treatment remains bone marrow allograft, but few patients are eligible for this therapeutic option due to their advanced age. For non-eligible MDS and AML patients, the conventional chemotherapy consists of demethylating agents, including azacytidine or decitabine [63,64,65], or high-doses of cytarabine. Unfortunately, most patients fail to respond durably to these drugs, and there is currently no effective second-line option. 

To adapt to the oxygen, nutrient, and glucose deprivation found during the different steps of tumor development, cancer cells modify their energetic needs through the fine-tuning of AMPK activity, a key metabolic sensor. As previously mentioned, AMPK is the master gene for the regulation of energy homeostasis. Its involvement in the regulation of apoptosis, autophagy and cell growth make this serine/threonine protein kinase a target of choice for the treatment of cancers in general and leukemia in particular [16,66,67]. Azacytidine-resistant MDS cell lines exhibited an altered response to apoptotic stimuli but displayed functional autophagy. Hence, triggering autophagy in resistant MDS cells induced autophagic cell death and bypassed the insensitivity to apoptosis [68,69]. Of note, it has recently been reported that the knockdown of *sf3b1*, a splicing factor mutated in 16% of MDS cases, deregulates the AMPK pathway [70]. This observation is of great interest since mutations of *sf3b1* are associated with good prognosis in MDS patients. Finally, cells issued from high-risk MDS patients exhibited a strong decrease of AMPK mRNA levels compared to those coming from low-risk MDS or healthy patients. This observation could explain the lower sensitivity of highly transformed MDS cells to variations in their energy environment.

### 6.2. Acute Myeloid Leukemia

The involvement of AMPK in the genesis and development of AML is well documented. Indeed, Saito et al. reported that leukemia initiating cells (LICs) responsible for the genesis of myeloid leukemia are protected from metabolic stress in the bone marrow through an AMPK-dependent mechanism [71]. This study clearly shows that dietary restriction induces metabolic cell survival. In contrast, depletion of AMPK expression in LIC reduces this cell population in the hypoxic bone marrow environment. Interestingly, human bone marrow stromal cells appear independent of the AMPK pathway, as knockout of AMPKα fails to sensitize these cells under dietary restriction or metformin treatment. Targeting AMPK depletes myeloid leukemia cells by disruption of glucose metabolism. In AML cell lines, exhibiting a high level of MAP kinase activation, AMPK activation was impaired. Indeed, Kawashima et al. demonstrated that glucose deprivation or metformin treatment could activate the AMPK pathway only in cells where ERK is weakly activated [72]. Moreover, in AML cells in which the MAP kinase pathway is overactivated, U0126, a MEK-specific inhibitor, was able to restore sustained AMPK activation under metformin treatment. The combination of an inhibitor of the MAPK pathway and an activator of the AMPK pathway leads to a significant decrease in cell growth and an increase in cell death in AML cells. This study suggests that both inhibition of an oncogenic pathway and activation of AMPK are necessary to trigger a strong anti-leukemic effect. The potent tumor suppressor role of the LKB1/AMPK pathway has been established in AML cells [73]. In this study, the authors showed that metformin activates the LKB1/AMPK pathway in AML and reduces tumor size in a mice xenograft model through the repression of mTOR-dependent oncogenic mRNA translation (c-MYC, CYCLIN-D, BCL-XL). It was recently demonstrated that co-activation of AMPK and mTORC1 could represent a good therapeutic strategy for AML [74]. Indeed, Sujobert et al. showed that GSK621, an AMPK direct activator, was highly cytotoxic for AML cells exhibiting a constitutive activation of mTORC1. As expected, this autophagy-dependent cell death induced by two concomitant signals was inhibited by rapamycin, an mTORC1 inhibitor.

Finally, although the strategy consisting of the use of AMPK agonists mimicking caloric restriction seems very promising, the development of an optimized and personalized therapy will need to block the predominant oncogenic pathway (overactivation due to mutations or translocation) and to activate AMPK in a concomitant manner. These complementary signals that will better impair cell growth and cell viability in AML will undoubtedly be a valuable option for patients in therapeutic failure.

### 6.3. Chronic Myelogenous Leukemia

Chronic myelogenous leukemia (CML) is a myeloproliferative syndrome linked to a hematopoietic stem cell disorder leading to increased production of granulocytes at all stages of differentiation. CML accounts for 15–20% of all cases of leukemia in adults [75] and is due to the t(9;22)(q34;q11) translocation, which encodes for the chimeric protein p210 BCR-ABL, a constitutively activated tyrosine kinase [76]. BCR-ABL expression leads to the engagement and activation of multiple pro-proliferative and anti-apoptotic cascades in transformed cells, including PI3K/AKT/mTOR and MAPK pathways [77,78,79]. Before the advent of targeted therapy, the gold standard for pharmacologic treatment of CML was α-interferon, but this treatment was associated with not-negligible toxicity and a median survival time of approximately five years [80]. In 2001, the identification of imatinib (Gleevec) as a small molecule ATP-pocket inhibitor of BCR-ABL dramatically re-defined the treatment of CML and had a major impact on the survival of patients with CML [81,82,83]. Imatinib mesylate along with second- (nilotinib, dasatinib, and bosutinib) and third-generation (ponatinib) tyrosine inhibitors (TKIs) have revolutionized the natural history of CML and have provided important treatment options for this leukemia that in the past was uniformly fatal [84]. Unfortunately, mutations rendering CML patients non-responsive to TKIs have been identified, including the threonine 315 to isoleucine (T315I) mutation and several others, which prevent binding of different TKIs to the active site of the ABL kinase, thereby avoiding its inhibition [85,86]. At present, more than 50 different mutation hotspots have been identified. BCR-ABL-independent mechanisms of resistance have also been reported to occur in tyrosine kinase inhibitor (TKI)-treated patients. In this regard, overexpression or hyperactivation of some members of the SRC family of kinases (LYN and HCK) have been described in cell lines and in some imatinib- and nilotinib-resistant patients [87,88,89]. Knowing that the PI3K/AKT/mTOR pathway is hyperactive in CML, indirect suppression of mTOR function by modulation of the AMPK pathway was proposed as an alternative therapeutic approach to overcome TKI resistances. Indeed, AMPK activation leads to mTOR inhibition through the phosphorylation and activation of the TSC1/2 complex [73,90] and/or a direct phosphorylation of the Raptor subunit on serines 722 and 792 [91], resulting in inactivation of the TORC1 complex. Most of the tested compounds that activate the AMPK pathway in the CML context are indirect activators. Among them, resveratrol, a naturally occurring substance found in grapes, triggers both apoptosis and autophagy in CML cells and is therefore able to overcome imatinib resistance [92,93]. Resveratrol-mediated autophagy is independent of BECLIN1 but mediated by a conjoint activation of AMPK and transcriptional upregulation of p62/SQSTM1 [93]. Interestingly, among the resistant cells that were sensitive to resveratrol, there were also cells expressing the T315I BCR-ABL mutant. Other studies have also pinpointed the anti-leukemic effects of AICAR or metformin on BCR-ABL transformed cells [12,60,94]. Unexpectedly, the effects of these compounds are often independent of AMPK activation. It has been established that the anti-leukemic effect of AICAR is dependent on protein kinase C-mediated autophagic cell death but independent of AMPK and apoptosis [12]. In conclusion, approaches to target cellular effectors of BCR-ABL, such as the PI3K/mTOR pathway, may provide alternative strategies to the use of TKIs to overcome resistance in refractory CML. The effect of direct AMPK activators on both TKI-sensitive and resistant CML cells has not been yet investigated.

### 6.4. Chronic Myelomonocytic Leukemia

**Figure 4:**
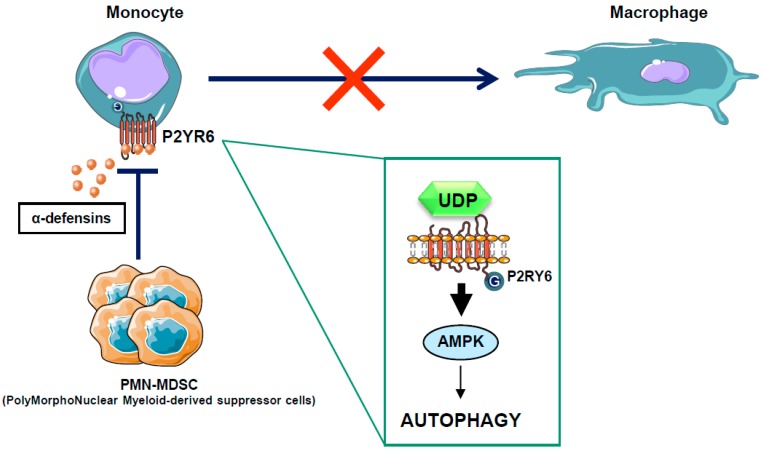
Mode of action for PMN-MDSC in the CMML context. In some CMML patients, PMN-MDSC secretes high levels of alpha-defensins, which antagonize the P2Y6 receptor, block autophagy activation, and inhibit the macrophagic differentiation of monocytes isolated from CMML patients. Alpha-defensin acts as a competitive inhibitor of UDP, the natural ligand of the P2Y6 receptor.

Chronic myelomonocytic leukemia (CMML) is a paradigmatic chronic myeloid malignancy that associates features of myelodysplastic syndromes (MDS) and myeloproliferative neoplasms (MPN) [95]. CMML is a clonal disease of the hematopoietic stem cell characterized by a persistent monocytosis (>1 × 10^9^/L), due to an accumulation of classical monocytes [96], and the aleatory presence of immature dysplastic granulocytes (PolyMorphoNuclear-MDSC or PMN-MDSC) in the peripheral blood of CMML patients [97]. These PMN-MDSCs that belong to the same clone as the leukemic monocytes appear to have immunosuppressive properties resembling those of the myeloid-derived suppressor cells (MDSCs), widely described in solid tumors. Whether these immature granulocytes contribute to autoimmune manifestations or immune-escape and progression of CMML is a conundrum and remains to be investigated. In recent years, large numbers of gene mutations have been discovered in CMML, none of which are specific to this condition, as they can be encountered with different frequencies in other myeloid neoplasms. These mutated genes encode signaling proteins (NRAS, KRAS, CBL, JAK2, FLT3, and several members of the Notch pathway), epigenetic regulators (TET2, ASXL1, EZH2, IDH1, and IDH2), and splicing factors (SF3B1, SRSF2, and ZRSF2) [98]. Mutations in the transcription regulators RUNX1, NPM1, and TP53 have also been reported in CMML. However, the role of these mutations in leukemogenesis is still unclear. Allogeneic stem cell transplantation is the only potentially curative option in patients suffering CMML, but it is associated with significant morbidity and mortality. Consequently, CMML patients are often treated like MDS patients with supportive care and hypomethylating agents, such as 5-azacitidine and decitabine, with overall response rates of 30–40% and complete remission rates of 7–17%, although with no impact on mutational allele burdens [99,100,101]. To improve their efficacy, azacytidine and decitabine have been combined with another drug, typically in “pick the winner” clinical trials with DNA-damaging drugs, immune-modulating drugs and histone deacetylase inhibitors, but none of these strategies has proven effective, and several were even found to be toxic [102,103]. There is therefore a strong need for alternative strategies aiming at increasing CMML patient overall survival. In a recent study, we have shown that CMML is characterized by defects in monocyte to macrophage differentiation [97]. These differentiation defects can be partly attributed to the presence of PMN-MDSCs that secrete high levels of alpha-defensins HNP1-3, which antagonize the purinergic receptor P2RY6 and inhibit AMPK-mediated autophagy in CMML patients [51]. Interestingly, we demonstrated that the physiological P2RY6 ligand UDP and the specific P2RY6 agonist MRS2693 can restore normal monocyte differentiation through re-induction of AMPK-dependent autophagy in primary myeloid cells from some, but not all, CMML patients (Figure 4). These results highlight an essential role for P2RY6-mediated autophagy through AMPK activation during the differentiation of human monocytes and pave the way for future therapeutic interventions for CMML. The use of direct AMPK activator in the CMML context could therefore be a promising therapeutic strategy.

## 7. AMPK Modulators for the Treatment of Hematopoietic Malignancies

Due to its implication at the crossroad of cellular metabolism and proliferation, AMPK has emerged as an attractive and promising target for a great number of human pathological situations, including metabolic diseases and cancer. Pharmacological compounds, such as 5-aminoImidazole-4-carboxamide-1-β-D-ribofuranoside (AICAR), metformin, and natural occurring compounds such as resveratrol and spermidine, activate AMPK by direct or indirect mechanisms. It has been reported that metformin, AICAR, or resveratrol exert potent antitumoral effects in both solid tumors and hematopoietic malignancies either sensitive or resistant to their treatment of reference [12,60,93,104,105,106]. However, the anti-leukemic effects of some but not all of these molecules were linked to AMPK activation, making some natural AMPK activators promising molecules for the treatment of hematopoietic malignancies. Due to the potential therapeutic benefit of AMPK modulators in numerous diseases, direct and more specific modulators of AMPK have been developed that mainly target the β subunits, and less frequently the α and γ subunits (please refer to [35,107] for detailed reviews). These inhibitors included β1-selective compounds such as A-769662, PF-06409577, and GSK621. A-769662 has been evaluated in different settings and although efficient, appeared to exhibit off-target effects and cannot be considered per se as a specific AMPK activator. MT47-100 is an allosteric modulator of AMPK that activates the AMPK complexes containing the β1 isoform and inhibits those comprising the β2 subunits (Table 1). All these inhibitors have been tested in different settings, including cancers, with variable efficacy. Among the direct activators of AMPK, only GSK621, which selectively binds to the β1 subunit, has been studied in hematopoietic malignancies. Indeed, it was reported that GSK621 selectively kills AML cell lines and AML primary cells, sparing normal hematopoietic progenitors [74]. The lethality of GSK621 was abrogated by chemical inhibition or genetic ablation of mTORC1, suggesting a preferential effect in AML cells with over-activation of the mTORC1 pathway. Finally, the GSK621 cytotoxicity in AML cells was strictly dependent on the eIF2α/ATF4 signaling pathway activated through mTORC1. Although it is premature to speculate that activation of AMPK could represent a new strategic therapy for all myeloid malignancies, these promising findings strongly suggest that specific activators of AMPKα1, such as GSK621, may represent a therapeutic opportunity in AML and likely more globally in cancers in which mTORC1 is over-activated.

## 8. Conclusions and Outlook

In this review, we have discussed how AMPK is involved and regulated during myeloid lineage differentiation and how it can impact myeloid cell pathophysiology. As autophagy is known to play an important role in the process of hematopoietic cell differentiation [108], it was expected that AMPK would be a regulator of this process. However, the role of the AMPK pathway as an important actor of the metabolic modifications necessary for myeloid differentiation is just emerging. As an energy-consuming process, physiological myeloid differentiation requires adaptation of AMPK expression and activity in part to sustain autophagy that is necessary for this process. Hematopoietic malignancies are systematically characterized by profound defects in cell differentiation. Restoration of an effective differentiation process in myeloid malignancies has thus emerged as a pertinent therapeutic strategy. This notion is particularly well exemplified by the successful use of arsenic trioxide to promote acute promyelomonocytic leukemia redifferentiation, even leading to cure in a majority of patients. Alteration of AMPK expression and/or activity is found in hematological malignancies such as CMML, and activation of the AMPK pathway appears to restore normal differentiation in some CMML patients [51]. Beside myeloid differentiation, recent evidence in the literature indicates that AMPK could also play a key role during normal erythroid cell differentiation (Ladli, M et al., 2018, Haematologica, in press). While using small molecule AMPK activators to treat a panel of human metabolic and neurodegenerative diseases now appears achievable, further investigations should be carried out before such activators, direct or indirect, reach the clinic for the treatment of myeloid malignancies.

## Figures and Tables

**Table 1:** AMPK activators include well-known pharmacological compounds, such as AICAR and metformin that act by increasing the AMP/ATP ratio, small molecules including A-769662, PF-06409577 and GSK621 that behave as AMPKβ1 subunit activator, and MT47-100, an AMPKβ1 activator and AMPKβ2 inhibitor. AMPK is also activated by a set of natural compounds including resveratrol and spermidine that indirectly increase the AMP/ATP ratio.

AMPK Activator	
Pharmacological Compounds	
AICAR	AMP:ATP ratio (up)
Metformin	
A-769662	AMPKβ1 subunit activators
PF-06409577	
GSK621	
MT47-100	AMPKβ1 subunit activator AMPKβ2 subunit inhibitor
**Natural Compounds**	
resveratrol	AMP:ATP ratio (up)
spermidine	
